# Wandering Spleen Complicated by Thrombocytopenia, Acute Appendicitis, and Sepsis: A Case Report and Literature Review

**DOI:** 10.3390/reports7030073

**Published:** 2024-09-02

**Authors:** Sri Inggriani, Callistus Bruce Henfry Sulay, Gilbert Sterling Octavius

**Affiliations:** 1 Abdominal Radiology, Department of Radiology, Faculty of Universitas Pelita Harapan, Tangerang 12930, Indonesia; 2 Medistra Hospital, Jakarta 12950, Indonesia; 3 Department of Radiology, Faculty of Universitas Pelita Harapan, Tangerang 15811, Indonesia

**Keywords:** wandering spleen, thrombocytopenia, appendicitis, Bochdalek hernia, sepsis

## Abstract

Wandering spleen (WS) is a rare condition often linked with torsion or infarction, but its association with Bochdalek hernia, acute appendicitis, and thrombocytopenia is exceptionally rare. We present a case of a nine-year-old girl who was admitted with acute abdominal pain, later diagnosed with WS, Bochdalek hernia, and acute appendicitis. A literature search was performed on PubMed and Google Scholar on 30 May 2024 with keywords including “Wandering spleen” and (“Bochdalek Hernia” OR “Sepsis” OR “Acute Appendicitis” OR “Thrombocytopenia”). The management was complicated by severe thrombocytopenia and post-operative sepsis, with *Klebsiella pneumoniae* as the causative agent. Imaging revealed an abnormally located spleen and significant splenic enlargement over time. The patient’s condition was managed non-operatively concerning the WS, avoiding splenectomy due to the risks of post-splenectomy sepsis. Instead, laparotomy was performed to address the appendicitis and diaphragmatic hernia. The patient experienced post-operative complications, including a seizure and persistent fever, which resolved with appropriate antibiotic therapy. This case underscores the complexity of managing WS with concurrent severe conditions, highlighting the importance of individualised treatment strategies. It also emphasises the need for further studies to explore optimal treatment modalities for such rare and complex presentations. This case serves as an educational example in clinical settings, demonstrating the challenges and considerations when treating multiple rare pathologies simultaneously.

## 1. Introduction

Wandering spleen (WS), also known as a displaced spleen, drifting spleen, floating spleen, pelvic spleen, splenic ptosis, splenoptosis, or systopic spleen, was first described by Van Horne in 1667 during an autopsy [1]. This condition is usually an incidental finding during routine imaging without any symptoms or of an acute abdomen during an emergency department visit [2].

In the past, the spleen was considered a dispensable organ, and splenectomy was considered the treatment of choice. However, it has long been discovered that the spleen has numerous physiological functions, such as hematopoiesis, iron metabolism, immune regulation, and red blood cell and platelet storage [3]. Since then, splenoplexy has been advocated when the spleen can still be salvaged [4].

This case report concerns a nine-year-old girl who presented with an acute abdomen, where the imaging finding showed WS, Bochdalek hernia, and acute appendicitis. This condition was complicated by post-operative sepsis. This case presents a teaching case in a clinical setting, and our case differs from the rest of the literature, which recommends surgery as the treatment of choice.

## 2. Methods

The literature search was performed on PubMed and Google Scholar on 30 May 2024 with keywords including “Wandering spleen” and (“Bochdalek Hernia” OR “Sepsis” OR “Acute Appendicitis” OR “Thrombocytopenia”). The MeSH terms were “wandering spleen” [MeSH Terms] OR (“wandering” [All Fields] AND “spleen” [All Fields]) OR “wandering spleen” [All Fields]) AND (“appendical” [All Fields] OR “appendicitis” [MeSH Terms] OR “appendicitis” [All Fields] OR (“hernias, diaphragmatic, congenital” [MeSH Terms] OR (“hernias” [All Fields] AND “diaphragmatic” [All Fields] AND “congenital” [All Fields]) OR “congenital diaphragmatic hernias” [All Fields] OR (“congenital” [All Fields] AND “diaphragmatic” [All Fields] AND “hernia” [All Fields]) OR “congenital diaphragmatic hernia” [All Fields]) OR (“thrombocytopaenia” [All Fields] OR “thrombocytopenia” [MeSH Terms] OR “thrombocytopenia” [All Fields] OR “thrombocytopenias” [All Fields])). A wandering spleen that is present after diaphragmatic hernia closure was not included in this review.

## 3. Case Presentation

A nine-year-old girl presented to the emergency department on the 26th of February with right lower abdominal pain for five days. She vomited three to five times a day, with a yellowish-green colour. Her highest temperature was 38 °C at home. She refused to eat and had very minimal fluid intake. She was born prematurely at eight months of gestation due to the premature rupture of membranes. She was also jaundiced at birth and currently has vision impairment, with severe tortuosity of the retinal arteries on fundoscopy.

**Figure 1:**
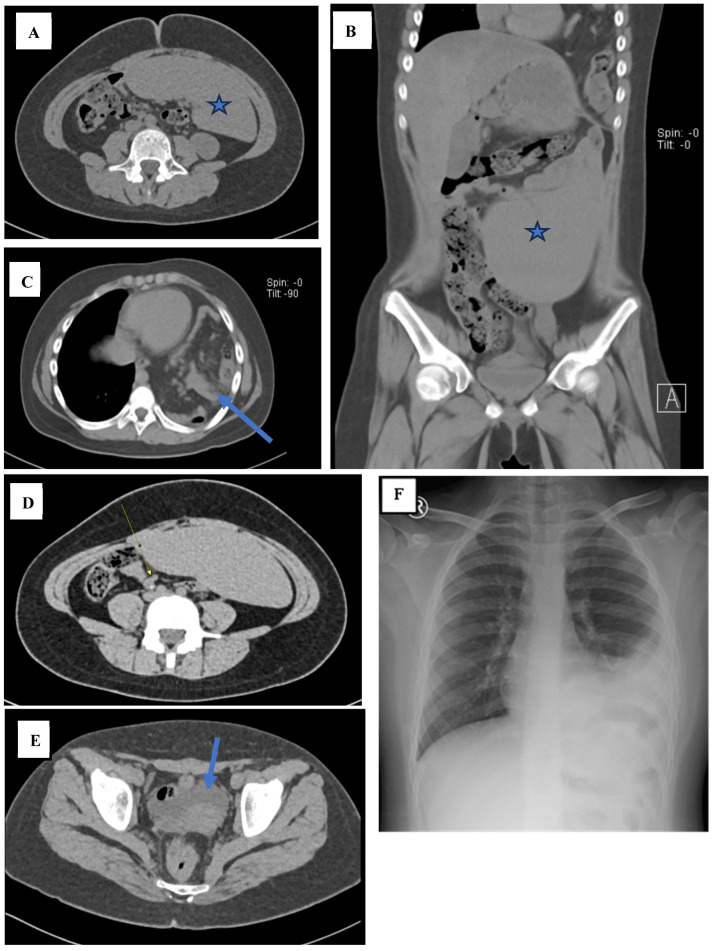
((**A**) axial; (**B**) coronal). The abdominal CT shows that the spleen was located at the mid-lower anterior abdomen, measuring ±14.3 × 18.6 × 5 cm (denoted by the star). (**C**) The components of the mesentery and intestines (arrow) are visible entering the left thoracic cavity through a defect in the left diaphragm measuring approximately 3.2 cm. (**D**) There is a tubular blind structure approximately 6.8 mm in diameter (arrow) with a hyperdense lesion inside measuring approximately 4.4 mm in diameter, extending approximately 57.7 mm in the lower right abdominal region. (**E**) Ascites is present in the pelvic region (arrow). (**F**) The pre-operative chest X-ray shows opacity in the basal left hemithorax and left paracardial region with opacification of the left diaphragm and a left costophrenic sinus.

On examination, she was relatively cooperative, with a Visual Analog Scale score of 2. There was a decrease in skin turgor, but she could still drink. The rest of the physical examination was unremarkable. She was obese, with a body mass index of more than the 95th percentile. The referring paediatrician suspected dengue fever with appendicitis and ordered a non-contrast abdominal computed tomography (CT). This showed that the spleen was located at the mid-lower anterior abdomen, measuring 14.3 × 18.6 × 5 cm with an estimated spleen index (ESI) of 1329.9, an estimated spleen volume (ESV) of 801.3 cm^3^, and estimated spleen weight (ESW) of 841.4 g (Figure 1A,B). The CT scan also showed a non-perforated, acute appendicitis with an appendicolith (Figure 1C), posterior left-sided Bochdalek diaphragmatic hernia (Figure 1D), and ascites at the pelvic region (Figure 1E). Her pre-operative chest X-ray showed pleuro-pneumonia (Figure 1F).

Initially, the white blood cell (WBC) count was slightly elevated at 10.9 × 10^3^/µL on the 27th of February, with a dramatic drop to a low of 2.61 × 10^3^/µL on 5th of March, followed by a sharp increase to 12.67 × 10^3^/µL by 6th of March, suggesting a fluctuating inflammatory response. Haemoglobin (Hb) levels steadily declined from 13 g/dL on the 26th of February to 9 g/dL by the 9th of March, indicating progressive anaemia, which might have reflected ongoing blood loss or bone marrow suppression. Platelet counts also showed a concerning trend, starting at 100 × 10^3^/µL on the 26th of February, falling to a critically low value of 18 × 10^3^/µL by the 8th of March, before a slight recovery to 172 × 10^3^/µL by the 14th of March, suggesting potential platelet consumption or sequestration, possibly related to splenic dysfunction. Notably, Procalcitonin (PCT) levels were significantly elevated at 284.75 ng/mL on the 6th of March, an indication of severe bacterial infection or sepsis, which later decreased to 33.07 ng/mL. The patient’s D-dimer levels were also markedly high at 5140 ng/mL on the 7th of March, hinting at significant coagulopathy, possibly due to systemic inflammation or splenic complications. Additionally, the ESR showed a sharp increase to 51 mm by the 7th of March, reinforcing the presence of ongoing inflammation. Urinalysis was remarkable for transient positive findings for ketones, erythrocytes, leucocytes, and nitrites on the 27th of February, which were later normalised, reflecting a possible transient urinary tract involvement or contamination. A full and detailed laboratory examination is available in Table S1.

A paediatric surgeon was consulted, and the girl underwent laparotomy for appendicitis and diaphragmatic repair, with the wandering spleen being managed non-operatively. There was an 8 cm left diaphragmatic defect with an incarcerated colon and trapped omentum. The omentum was resected, while the colon was released without any colectomy. The diaphragm was sutured with an interrupted technique. Neither splenectomy nor splenopexy was performed. The operation was conducted on the same day as the CT scan, and it took 2 h and 45 min with minimal intra-operative bleeding of less than 50 mL. She received ceftriaxone as the post-operative antibiotic. No intra-abdominal drain was placed.

The girl was monitored for fever, pain, and spontaneous defaecation. The pain score slowly improved over time with the use of epidural analgesia, and there was spontaneous defaecation on the fifth post-operative day. There was no fever post-operatively until the 4th of March. On the 5th of March, she experienced a tonic–clonic seizure for around one minute. Her temperature was 41.5 °C at that time. The seizure was terminated with 10 mg of diazepam per rectal. After seizure termination, her blood pressure was 111/55 mmHg, her pulse was 150 per minute, and her pulse oximetry reading was 100% on three litres per minute of oxygen via nasal cannula. Despite the use of antibiotics and antipyretics, the fever did not subside. A blood culture was ordered on the 8th of March, and the result was available on the 11th of March. The culture was positive for *Klebsiella pneumoniae*, which was sensitive to amoxicillin/clavulanate, piperacillin/tazobactam, ertapenem, meropenem, imipenem, levofloxacin, gentamicin, amikacin, and cotrimoxazole. This explained why the infection did not subside with ceftriaxone, as the organism was resistant to ceftriaxone. Before ordering the blood culture, ceftriaxone was substituted with meropenem three times daily with amikacin. Based on the blood culture and clinical grounds, a diagnosis of sepsis was made. On the same day, a peripheral blood smear showed anisocytosis, hypochromic microcytic anaemia, with pencil cell morphology. Since the patient also showed persistent thrombocytopenia, the clinician ordered 8 units (400 mL) of thrombocyte concentrate (TC). Her blood group was A (+). The blood results were negative for the malaria panel.

**Figure 2:**
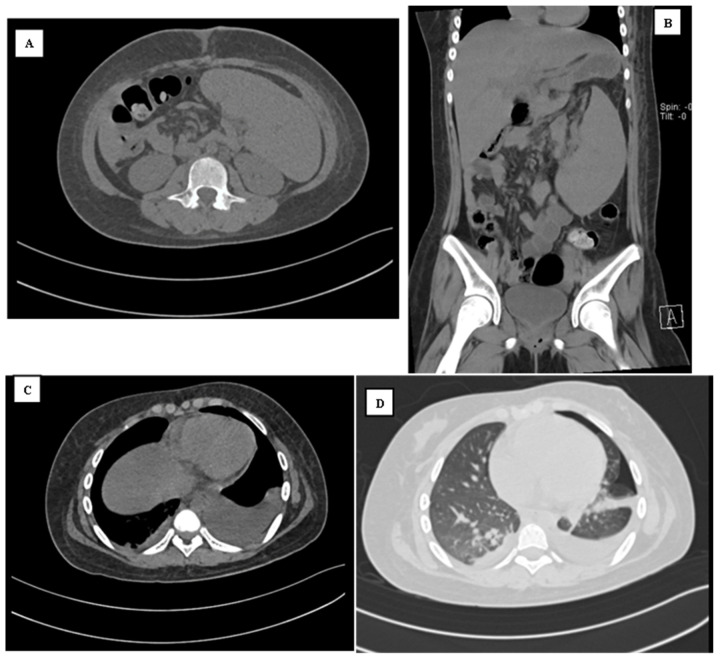
((**A**) axial; (**B**) coronal). The abdominal CT shows that the spleen was located at the mid-lower anterior abdomen, measuring ±14 × 16.7 × 7 cm, which is relatively similar compared to the last CT scan. (**C**) There is bilateral pleural effusion, which was more severe on the left and (**D**) the lung window shows the presence of left pneumothorax.

On the 8th of March, a second non-contrast abdominal CT scan was performed. The wandering spleen was relatively similar compared to the previous CT scan, measuring 14 × 16.7 × 7 cm with an ESI of 1870.4, an ESV of 1114.8 cm^3^, and an ESW of 1170.5 g (Figure 2A,B). There was bilateral pleural effusion, more severe on the left side (Figure 2C), and left-sided pneumothorax (Figure 2D). Since the patient did not complain of any dyspnoea, the pneumothorax was managed conservatively. The fever completely subsided on the 12th March. There was no clinical deterioration in between the observation period. The patient was discharged on the 14th March, with a total length of stay of 16 days.

## 4. Discussion

The range of clinical manifestations of wandering spleen (WS) varies greatly, from being asymptomatic and discovered incidentally on imaging to causing acute or chronic abdominal pain due to torsion and volvulus. There have even been reports of spontaneous rupture [5]. It is difficult to pinpoint exactly if WS contributes to abdominal pain in this patient as two other conditions may also explain abdominal discomfort. This lack of contrast also does not help to distinguish whether there are signs of infarction, such as a twisted pedicle or the whirl sign [6]. However, the spleen became progressively enlarged on the second CT imaging, presumably due to the edematous nature of organ failure related to sepsis. Although no follow-up imaging was performed, the absence of emergency return due to abdominal discomfort may point to the fact that the WS was an incidental finding at that time. However, it should also be noted that absence of return does not mean evidence of absence.

The literature search resulted in 31 articles, with five articles [7,8,9,10,11,12] being excluded due to the presence of WS after diaphragmatic repair. The rest of the 21 articles were excluded on the basis that it is not associated with appendicitis, congenital diaphragmatic hernia, or thrombocytopenia. Hence, five articles were selected, with two articles associated with WS and CDH [12,13], two articles associated with WS and thrombocytopenia [14,15], and one article associated with WS and appendicitis [16] (Table 1).

The aetiology of WS has been thought to be congenital (with the laxity of the gastrosplenic ligament), and acquired causes have been due to malaria or malignancies [17]. In our patient, congenital theory has a strong association as the patient was born prematurely. This association is further supported by the presence of a Bochdalek hernia in this patient. The concurrence of both conditions is rare, although it has been reported before in paediatric patients. There are other congenital diseases associated with WS in children, such as prune belly syndrome, gastric volvulus, renal agenesis, and diaphragmatic eventration [18]. Acute appendicitis has no direct correlation with WS, and these three findings together in one patient can be considered a very rare finding [16].

The causes of thrombocytopenia are vast, and pointing out the exact aetiology requires a clinical correlation between medical history records, physical findings, other laboratory results, and radiology findings. Indonesia still has a high endemicity for dengue fever, and thus, the top differential diagnosis includes dengue fever [19]. However, despite checking the antigen and antibody towards the dengue virus, none returned positive. The infusion of TC also did not help to increase the platelet count in the short term. Hence, the cause of thrombocytopenia may be due to an anatomical cause, which is the WS, due to hypersplenism [14].

Splenoplexy or splenectomy, performed via laparotomy or laparoscopy, has been the most commonly mentioned surgical treatment [20]. The criteria for surgery are not clear. The advantages and disadvantages between different methods of surgeries are also dubious at best, as the evidence is derived mostly from case reports. Most authors suggest surgeries for all cases of wandering spleen, regardless of the symptomatology upon presentation [21,22]. However, this case proves that not all wandering spleens should be executed. A prospective cohort study comparing the benefits of operative vs. non-operative treatment would be ideal, but due to the rarity of this case, this approach is almost impossible. The microbiological cause of sepsis in this case was *Klebsiella pneumoniae* ssp. Pneumoniae is an encapsulated bacterium that can only be cleared immunologically with the help of the spleen [22,23]. One of the more serious side effects of splenectomy is post-splenectomy sepsis, with a 30–60% mortality rate [21]. This case presents a clinical dilemma where if the spleen was removed, as suggested by the literature, death was a very possible outcome. Long-term follow-up is needed for all patients with wandering spleen who are not operated on to determine the progressive nature of this disease.

## 5. Conclusions

This case report highlights the complexity of managing the wandering spleen (WS), particularly when associated with concurrent conditions, such as Bochdalek hernia, acute appendicitis, and post-operative sepsis. Despite the general recommendation in the literature to perform splenectomy or splenopexy for WS, this case demonstrates that a non-operative approach can be successful, even in the presence of sepsis caused by *Klebsiella pneumoniae*. The patient’s outcome underscores the importance of individualised treatment plans, considering the potential risks of surgery, including post-splenectomy sepsis, against the benefits of spleen preservation.

Long-term follow-up is essential for patients with untreated WS to monitor for potential complications. This case contributes to the limited evidence suggesting that conservative management may be a viable option in selected cases, challenging the prevailing view that all cases of WS should undergo surgical intervention.

## Figures and Tables

**Table 1:** Summary of related studies in the literature.

Author (Year)	Sex and Age (Year)	Associated Comorbidities	Surgery	Outcome
Dangen (2020) [13]	F, 17	CDH and splenic torsion	Closure of hernia and splenectomy	Post-operative pneumothorax but recovered well
Mirkes (2011) [14]	F. 21 (16 weeks pregnant)	Thrombocytopenia	None	Low platelet even after delivery
Zhao (2022) [16]	M, 18	Appendicitis and splenic torsion	Laparoscopic removal of spleen and appendix	Discharged seven days after surgery
Pelizzo (2001) [12]	F, 12	CDH	Diaphragmatic repair with simple medial dislocation of the spleen	Recovered well
Moll (1996) [15]	F, 30	Thrombocytopenia	Splenectomy	Recovered well and no longer has thrombocytopenia

CDH, congenital diaphragmatic hernia.

## Data Availability

The data presented in this study cannot be shared publicly due to privacy concerns. The data will be shared upon reasonable request to the corresponding author.

## Supplementary Materials

The following supporting information can be downloaded at https://www.mdpi.com/article/10.3390/reports7030073/s1. Table S1: Serial laboratory values during admission.
