# Supplementary material for: Wandering Spleen Complicated by Thrombocytopenia, Acute Appendicitis, and Sepsis: A Case Report and Literature Review
Source: Reports (MDPI). 2024 Sep 2;7(3):73. doi: 10.3390/reports7030073 (PMC12225254; doi:10.3390/reports7030073)
Supplement: Supplementary file 1 [file reports-07-00073-s001.zip › reports-3167058-supplementary.pdf]

Table S1. Serial laboratory values during admission.

Laboratory parameters*	26/02	27/02	02/03	04/03	05/03 (06:18)	05/03 (17:36)	06/03 (05:55)	06/03 (16:54)	07/03 (05:35)	07/03 (17:44)	08/03 (07:10)	08/03 (15:40)	09/03	11/03	14/03
Hb (g/dL) (12-16)	13	12.5		10.7	10.6	10.3	9.5	10.5	9.5	9	9.2	9.3		9	9.3
WBC (10^3/μl) (5-13)	6.63	10.9		4.45	2.61	5.06	10.81	12.67	7.76	5.37	5.12	12.4		8.34	7.38
Basophil (%) (0-1)	0			0							0	0		0	0
Eosinophil (%) (0-4)	0			2							0	0		1	1
Band neutrophil (%) (2-6)	0			0							3	3		1	2
Segmented neutrophil (%) (32-52)	85			80							76	70		68	43
Lymphocyte (%) (30-60)	11			15							15	19		22	42
Monocyte (%) (2-8)	4			3							6	8		8	12
NLR	7.73			5.33							5.27			3.14	1.07
ESR (mm) (0-10)	5			26							51			24	26
Erythrocyte (10^6/μl) (4-5.3)	6.31	6.01		5.19	5.1	5.05	4.61	5.03	4.67	4.41	4.55	4.57		4.39	4.64
Ht (%) (35-45)	40.9	38.6		33.1	32	32.6	29.2	31.8	29	27.4	28.4	28.3		26.6	29.2
MCV (fL) (75-91)	64.8	64.2		63.8	62.7	64.6	63.3	63.2	62.1	62.1	62.1	61.9		60.6	62.9
MCH (pg) (25-33)	20.6	20.8		20.6	20.8	20.4	20.6	20.9	20.3	20.4	20.4	20.4		20.5	20
MCHC (g/L)	31.8	32.4		32.3	33.1	31.6	32.5	33	32.8	32.8	32.4	32.9		33.8	31.8
Platelet (10^3/μl) (150-400)	100	105		118	71	48	33	31	20	22	18	19		61	172
PT (seconds)															
Control	10.9														
Patient (9.3-11.4)	11.1														
INR	1.02														
APTT (seconds)															
Control	23.7														
Patient (23.4-31.5)	28.9														
ALP (U/L) (<500)	189														
AST (U/L) (5-34)	16								56	64					
ALT (U/L) (<55)	7								31	33					
Na (mmol/L) (135-145)	136	135		136									135		

K (mmol/L) (3.5-5.1)	4.5	4.3		4.5		4.2
Cl (mmol/L) (97-111)		102		103		
Ca (mg/dL) (8.4-10.2)	9.4					7.6
Amylase (U/L) (25-125)	56					
Lipase (U/L) (8-78)	9					
CRP (mg/L) (<5)	1.9					
Albumin (g/dL) (3.5-5)	4.6					2.5
Dengue antigen NS1		Negative		Negative	Negative	
Urinalysis						
Color (yellow)		Dark yellow	Light yellow			
Clearness (clear)		Turbid	Clear			
Albumin (negative)		(+) 1	Negative			
Glucose (negative)		Negative	Negative			
Erythrocyte sediments (/µl) (<3)		35	1			
Leucocyte sediments (/µl) (<10)		15	2			
Epithelial cells		(+)	(+)			
Crystals (negative)		Negative	Negative			
Others		Positive for bacteria	Negative			
pH (4.5-8)		6	7			
SG (1,000-1,030)		1,020	1,004			
Bilirubin (negative)		Negative	Negative			
Urobilinogen (mg/dL) (0.1-0.9)		1	Birnak			

Keton (Negative)	Positive	Positive				
Erythrocyte (Negative)	(+) 3	Negative				
Leucocyte (Negative)	(+) 1	Negative				
Nitrite (negative)	Positive	Negative				
Anti-Dengue IgG			Negative		Negative	
Anti-Dengue IgM			Negative		Negative	
D-Dimer (ng/mL) (<500)				5140		
PCT (ng/mL) (<0.05)				284.75		33.07
Interferon-gamma release assay					Negative	
Faeces analysis					Positive for occult blood (Hb)	

*The normal values are indicated in brackets while the bold numbers are the abnormal values
Hb, Hemoglobin; WBC, White blood cell; NLR, Neutrophil lymphocyte ratio; ESR, Erythrocyte sedimentation rate; MCV, Mean corpuscular volume; MCH, Mean corpuscular hemoglobin; MCHC, Mean corpuscular hemoglobin concentration; PT, Prothrombin time, APTT, Activated partial thromboplastin clotting time; ALP, Alkaline phosphatase; AST, Aspartate aminotransferase; ALT, Alanine aminotransferase; Na, Sodium; K, Potassium; Cl, Chloride; Ca, Calcium; CRP, C-Reactive protein; Ht, Hematocrit; SG, Specific gravity; IgM, Immunoglobulin M; IgG, Immunoglobulin G; PCT, Procalcitonin
